# Association of State Firearm Laws With Firearm Ownership and Mortality

**DOI:** 10.1016/j.focus.2024.100250

**Published:** 2024-06-20

**Authors:** Roni Barak Ventura, James Macinko, Manuel Ruiz Marín, Maurizio Porfiri

**Affiliations:** 1 Center for Urban Science + Progress, New York University Tandon School of Engineering, Brooklyn, New York; 2 Department of Mechanical and Aerospace Engineering, New York University Tandon School of Engineering, Brooklyn, New York; 3 Department of Community Health Sciences, Fielding School of Public Health, University of California, Los Angeles, Los Angeles, California; 4 Department of Health Policy and Management, Fielding School of Public Health, University of California, Los Angeles, Los Angeles, California; 5 Department of Quantitative Methods, Law and Modern Languages, Technical University of Cartagena, Cartagena, Spain; 6 Murcia Bio-Health Institute, Health Science Campus, Murcia, Spain; 7 Department of Biomedical Engineering, New York University Tandon School of Engineering, Brooklyn, New York

## Abstract

**Introduction:**

Firearm injury is a leading cause of death among Americans. Because the right to bear arms is protected by the Second Amendment, policymakers must consider the impact of legislation on both firearm ownership and firearm harms. The current state of knowledge in firearm research majorly examines the impact of firearm legislation on firearm injuries and fatalities alone, and it relies on correlational analyses. The few studies that consider causal effects employ counterfactual-based inference. This study introduces information-theoretic tools to explore the role of firearm laws in mitigating firearm harms while maintaining citizens’ right to bear arms.

**Methods:**

The authors study monthly time series from January 2000 to October 2019 for the implementation of firearm laws from RAND's State Firearm Law Database, firearm deaths by intent from the Centers for Disease Control and Prevention databases, and firearm ownership from an econometric model. The authors employ transfer entropy, an information-theoretic method that relies on Granger causality, to infer relationships from time series. Specifically, the authors examine transfer entropy from firearm restrictiveness to deaths per firearm owner, firearm ownership, and firearm deaths, independently.

**Results:**

On a national level, the authors uncover a negative association from firearm restrictiveness to deaths per firearm owner and a positive association from firearm restrictiveness to firearm ownership. On a regional level, the authors identify a negative association from firearm restrictiveness to deaths per firearm owner in the Northeast, a negative association from firearm restrictiveness to firearm ownership in the Midwest, and a negative association from firearm restrictiveness to firearm suicides in the South.

**Conclusions:**

The authors present an information-theoretic approach to study relationships in firearm research. This method provides preliminary evidence for the role of restrictive legislation in promoting safe firearm ownership. The authors find that firearm acquisition considerably increases after the implementation of restrictive firearm laws, and simultaneously, firearm deaths decrease. These effects vary with respect to death by intent and the geographic region the laws were implemented in.

## HIGHLIGHTS

• It remains unclear whether firearm legislation promotes safer firearm ownership.
• Transfer entropy is computed from firearm legislation to deaths per firearm owner.
• Restrictive legislation increases firearm ownership and reduces firearm mortality.
• The result suggests that people panic-buy firearms before restrictive laws are implemented.

## INTRODUCTION

Firearm violence is a major public health issue in the U.S., where rates of firearm injury are among the highest in the world and steadily increasing.^1^ In 2020, more than 45,000 Americans died by firearms, averaging 123 deaths per day and surpassing the number of deaths due to motor vehicle crashes.^2^ Although official data on mortality in 2022 are yet to be released, provisional estimates suggest that this figure climbed to 48,000 and an average of 132 fatalities per day.^3^ These grim statistics are tightly connected to firearm ubiquity, whereby literature consistently correlated firearms accessibility with firearm harms.^4^, ^5^, ^6^, ^7^, ^8^ Government authorities often act to regulate harmful agents; yet, there is no consistent approach to firearm regulation among U.S. states. This may stem from the fact that firearms play a defining role in American culture and identity,^9^ so that many Americans continue to bear arms.^10^ Thus, to reconcile citizens’ desire to bear arms with the eminent need to mitigate risks of firearm injury, policymakers must identify legislative interventions that minimize harms without limiting firearm acquisition.

Although numerous studies provide insights into the potential of firearm legislation in reducing firearm harms, they largely rely on correlational analyses, and only a few employ counterfactual-based causal inference methods,^11^, ^12^, ^13^, ^14^, ^15^, ^16^, ^17^, ^18^, ^19^, ^20^ likely owing to lack of federal funding and unavailability of data on firearm ownership and harms.^21^, ^22^, ^23^ Causal inference with counterfactuals is limited in the study of state firearm legislation and presently cannot provide definitive evidence for the effectiveness of firearm laws.^24^, ^25^, ^26^ In particular, counterfactual approaches require the identification or design of appropriate untreated units to compare with. In many instances, control units would encompass vastly different characteristics in terms of demographics, culture, and political ideology such that a comparison may not be appropriate, even with covariate adjustment.^24^ Furthermore, because all U.S. states have implemented a firearm law of some kind, the extent to which some states should be considered treated against others is difficult to assess. In this context, information theory emerges as a powerful means to complement counterfactual-based methods in the inference of associations from time series. At the heart of information theory lies the notion of entropy, which quantifies uncertainty with respect to a measurement.^27^^,^^28^ Transfer entropy (TE) quantifies temporal associations in a Granger sense as reduction in the uncertainty of predicting the future state of a process from its present, given additional knowledge about the present or past of another process.^29^ Although model-free inference of temporal associations based on TE has some limitations (including sensitivity to unmeasured variables),^30^ it does not require the identification of untreated units and therefore overcomes some shortcomings of counterfactual-based inference in firearm research.

In this study, the authors employ TE to explore the impacts of permissive and restrictive firearm legislation on firearm ownership and firearm harms simultaneously. In a country with inhabitants who revere the right to bear arms, effective and widely adopted firearm legislation must consider its influence on both outcomes. To this end, the authors combine the measures of firearm deaths and firearm ownership into a quantity called deaths per firearm owner to capture the safety of owning a firearm. The authors test 3 hypotheses in 3 independent analyses, considering all firearm law classes. First, the authors test the hypothesis that deaths per firearm owner will improve upon the implementation of restrictive laws and compromised upon the implementation of permissive laws. This analysis is motivated by a multitude of studies that hint at such effects.^31^, ^32^, ^33^, ^34^, ^35^, ^36^, ^37^ The second hypothesis addresses the intent of injury (accidents versus homicides versus suicides). Laws often target aspects of regulation that are relevant for one intent of injury and not another.^20^^,^^35^^,^^38^^,^^39^ Therefore, the authors hypothesize that restrictive (permissive) firearm laws will influence firearm accidents, homicides, and suicides differently. The third hypothesis examines the 2 elements that define deaths per firearm owner: firearm violence and firearm ownership. In agreement with the first hypothesis, the authors expect that restrictive (permissive) legislation will reduce (increase) firearm violence. At the same time, on the basis of findings that media coverage of firearm legislation leads to surges in firearm acquisition,^40^, ^41^, ^42^ the authors anticipate that firearm ownership will not be negatively affected by legislation.

Data on firearm deaths and firearm ownership are available on a state level. However, state firearm laws are not implemented with sufficient frequency that would support statistically robust TE analysis. Therefore, the authors study the 3 hypotheses mentioned earlier on a national level as well as in each U.S. region. The authors hypothesize that results would vary among regions owing to differences in culture, demographics, and political orientation.

## METHODS

### Study Sample

To test the hypotheses put forth, the authors collected data on 3 variables: firearm laws, firearm deaths, and firearm ownership. Data were collected for each month between January 2000 and October 2019. Because data on firearm ownership were missing for Alaska, Hawaii, and District of Columbia, these states were excluded from the study.

Data on firearm laws were obtained from RAND's State Firearm Law Database.^43^^,^^44^ This database contains information about firearm-related laws, including the U.S. states they were passed in, the dates they became effective, and a summary of their content. Importantly, the database systematically categorizes each law into 1 of 20 different law classes and denotes its overall effect (permissive or restrictive) on the basis of the legal regime they impart relative to the one prior to their implementation.^44^ A law that eases access to and use of firearms is considered permissive, whereas a law that curtails access to and use of firearms is categorized as restrictive. Within the time period under consideration, the database contains law changes from all 20 law classes, including 30 actions related to stand-your-ground laws (all of which are considered permissive), 5 actions related to child access (all of which are restrictive), and 43 actions related to background checks (38 restrictive new implementations or modifications, 4 permissive repeals, and 1 permissive modification).

Data on firearm deaths were collected from the Centers for Disease Control and Prevention WONDER database.^2^ WONDER reports death rates of U.S. residents on the basis of their death certificates. In particular, the database allows to subset death rates on the basis of injury intent (homicide, suicide, or unintentional) and mechanism (firearm or otherwise). For each intent, the authors grouped the results by state, year, and month and obtained a monthly time series. In addition, the authors created a time series of firearm deaths by summing the 3 injury intents.

Data on firearm ownership were based on measurements from a spatiotemporal econometric model.^42^ This model estimates the monthly fraction of firearm owners out of the population by integrating 2 cogent proxies, background checks per capita and fraction of suicides committed with a firearm, and calibrating on yearly survey data that assess the fraction of the population that can access a firearm in their home or property. Unlike other proxies, this model accounts for geographic spillover effects whereby firearms move across state borders and incorporates temporal autoregression. Authors also estimated the number of firearm owners in a given U.S. state and month by multiplying the model's output by the state's population size in the same year (taken from the U.S. Census Bureau^45^). Such an estimate is not exact because it maps 1 owner to 1 firearm.

For all 3 variables, the authors generated time series on state level. State-level time series were then aggregated following the U.S. Census Bureau designations^46^ to obtain time series for each variable in each region (Appendix Section 1, available online).

### Measures

From the data that were collected, the authors generated 2 additional time series: firearm restrictiveness and deaths per firearm owner. For firearm restrictiveness, the authors created a continuous monthly time series that contained the cumulative number of permissive legislative actions that were implemented since January 1, 2000, subtracted from the cumulative number of restrictive legislative actions in the same period. Because each implemented law impacts a fraction of the nation, the authors scaled each by the fraction of the population of the entire U.S. population affected by the law.

For deaths per firearm owner, the authors divided firearm deaths by firearm ownership. To evaluate the influence of firearm restrictiveness on firearm death by intent, the authors also generated time series for each injury intent: accidents per firearm owner, homicides per firearm owner, and suicides per firearm owner. Deaths caused by legal interventions or undetermined causes were not included in those counts.

Finally, to study interactions on a regional level, the authors generated time series of firearm restrictiveness, firearm deaths, firearm ownership, and deaths per firearm owner for each of the 4 U.S. regions: Northeast, Midwest, South, and West (Appendix Section 1, available online).

### Analysis

The authors performed 3 analyses with TE on a national level (Appendix Section 2, available online). To test the first hypothesis, the authors computed TE from firearm restrictiveness to deaths per firearm owner. In Appendix Section 3 (available online), the authors tested alternative measures of deaths per firearm owner where popular measures of firearm prevalence are used. To test the second hypothesis, the authors computed TE from firearm restrictiveness to accidents per firearm owner, homicides per firearm owner, and suicides per firearm owner. To test the third hypothesis, the authors computed TE independently from firearm restrictiveness to firearm ownership, firearm deaths, firearm accidents, firearm homicides, and firearm suicides. In total, TE was computed for 9 relationships on a national level. All relationships were evaluated with delays ranging from 0 to 11 months. To account for multiple comparisons in the delay analysis, levels of significance were corrected through false discovery rate.^47^ All TE values and associated statistics are reported in Appendix Section 4 (available online). The Results section of this article summarizes TE values that remained statistically significant after this correction, although the authors highlight that the absence of a statistically significant effect does not imply that an association does not exist. To explore the possibility of reverse causal effects from outcomes to firearm restrictiveness, in Appendix Section 5 (available online), the authors performed an equivalent TE analysis where firearm restrictiveness is the target variable, and deaths per firearm owner, firearm ownership, and firearm deaths are the source variables.

To pinpoint trends on a regional level, the authors selected the delay associated with the largest amount of TE on a national level and performed TE analysis in each of the 4 regions. The authors computed TE from firearm restrictiveness to deaths per firearm owner, firearm ownership, and firearm suicides.

Data analysis was performed between May 2023 and February 2024. All analyses were performed in MATLAB (MATLAB R2022b, The MathWorks, Inc., Natick, MA) with a significance level α=0.05 (α=0.10 was used for assessing trends).

## RESULTS

**Figure 1:**
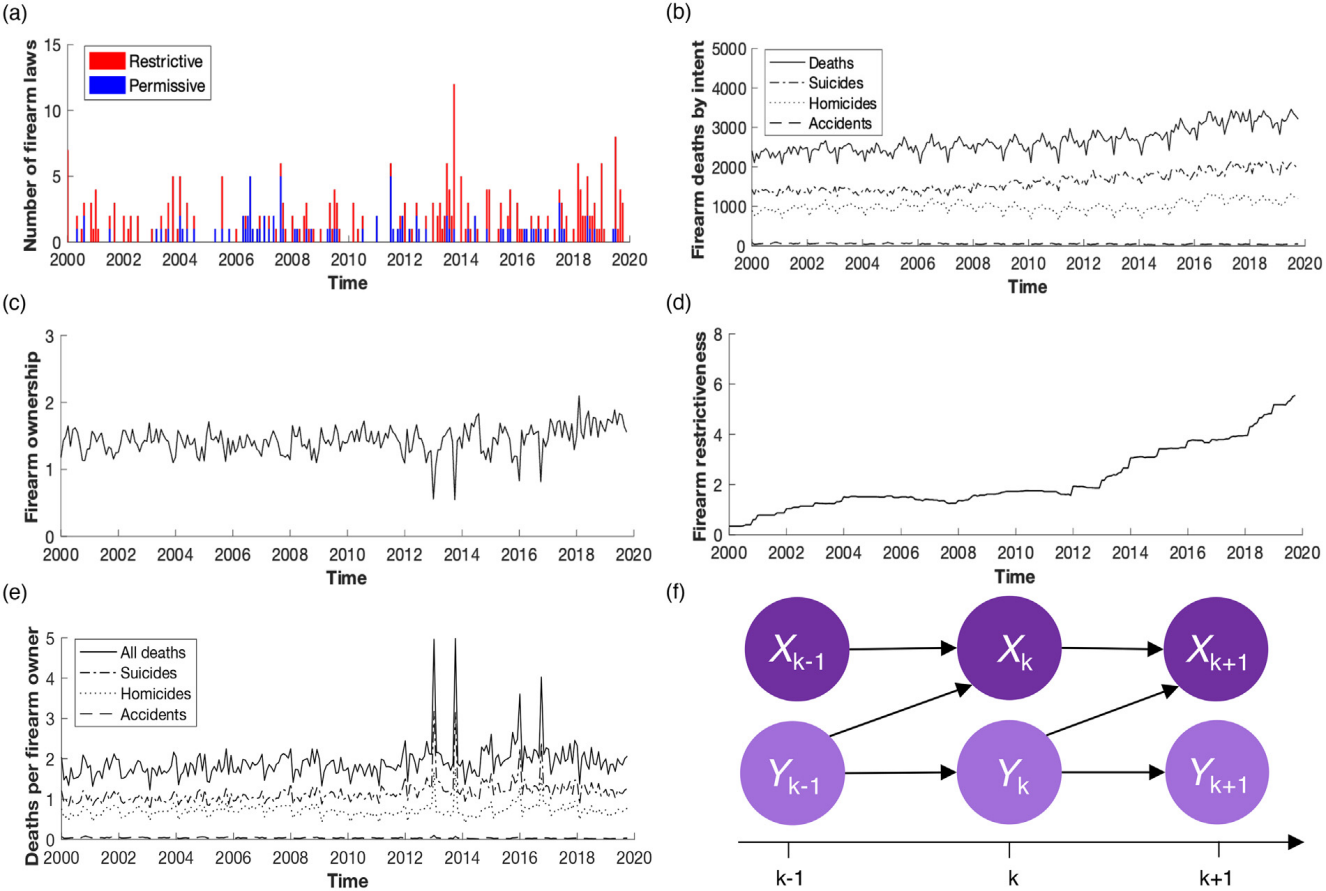
Analysis of national times series in this study. (a) Stacked number of restrictive (red) and permissive (blue) firearm laws. (b) Number of firearm deaths (solid), disaggregated by intent into accidents (dashed), homicides (dotted), and suicides (dash dotted). (c) Firearm ownership, reflecting the number of firearms in the entire country. (d) Firearm restrictiveness. (e) Deaths per firearm (solid), divided by intent into accidents (dashed), homicides (dotted), and suicides (dash dotted). (f) Illustration of transfer entropy from Y to X. Should Y help to predict X continuously throughout the time series, the relationship between the 2 variables will be deemed causal.

On a national level, a total of 318 nonredundant laws were recorded: 222 restrictive and 96 permissive (Figure 1a). The time series of firearm deaths was majorly composed of firearm homicides and suicides rather than firearm accidents (Figure 1b). In the specified time period, 11,844 accidents; 239,753 homicides; and 385,651 suicides were recorded, totaling 637,248 deaths by firearms. The time series of firearm ownership showed that the number of firearm owners in the U.S. ranged from 54.7 million to 209 million (Figure 1c). Processed time series (firearm restrictiveness and deaths per firearm owner) were also constructed. Firearm restrictiveness consisted of 127 changes and generally increased over time (Figure 1d). Deaths per firearm owner were predominantly driven by suicides, followed by homicides and accidents (Figure 1e). Regional time series are presented in Appendix Section 1 (available online).

**Figure 2:**
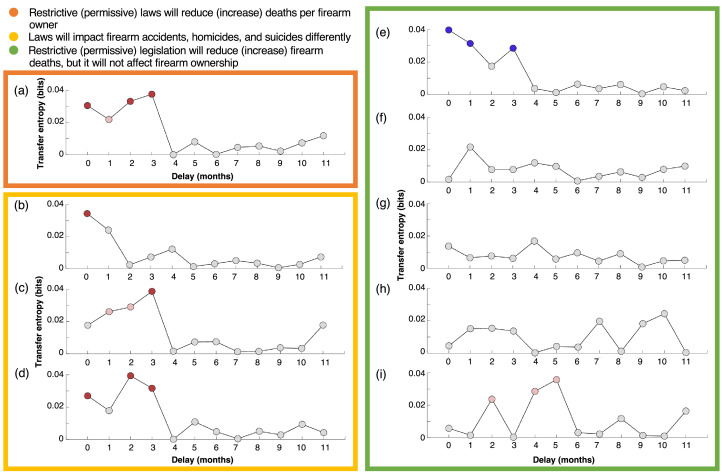
Addressing the 3 hypotheses on a national level. (a) In the orange frame, the first hypothesis is tested by computing transfer entropy from firearm restrictiveness to deaths per firearm. In the yellow frame, the second hypothesis is tested by computing transfer entropy from firearm restrictiveness to (b) accidents per firearm, (c) homicides per firearm, and (d) suicides per firearm. In the green frame, the third hypothesis is tested by computing transfer entropy from firearm restrictiveness to (e) firearm ownership, (f) firearm deaths, (g) firearm accidents, (h) firearm homicides, and (i) firearm suicides. Gray circles indicate that transfer entropy was not significantly different from 0 in permutation tests. Red and pink circles indicate a negative association that is different from 0 with a significance level of α=0.05 and α=0.01, respectively. Blue circles indicate a positive association that is different from 0 with a significance level of α=0.05.

TE from firearm restrictiveness to deaths per firearm owner revealed negative associations for delays of 0, 1, 2, and 3 months (TE=0.031, 0.022, 0.033, and 0.038 bits, respectively, and ρ= −0.239, −0.271, −0.371, and −0.271, respectively) (Figure 2a). TE was significantly different from 0 for delays of 0, 2, and 3 months (*p*=0.007, 0.005, and 0.003, respectively) and marginally different from 0 for delay of 1 month (*p*=0.030). The values of TE, *p*-values of permutation tests, and partial correlation coefficients for the remaining delays are reported in Appendix Section 3 (available online).

Results for TE from firearm restrictiveness to the disaggregated forms of deaths per firearm owner are reported in Figure 2b–d and Appendix Table 3 (available online). TE to accidents per firearm owner revealed a negative association with a delay of 0 months that is significantly different from 0 (TE=0.034 bits, *p*=0.004, and ρ= −0.211). When considering homicides per firearm owner, 3 negative associations emerged for delays of 1, 2, and 3 months (TE=0.026, 0.029, and 0.039 bits, respectively, and ρ= −0.268, −0.358, and −0.249, respectively). TE was significantly different from 0 for a delay of 3 months (*p*=0.002) and marginally different from 0 for delays of 1 and 2 months (*p*=0.015 and 0.010, respectively). TE to suicides per firearm owner also yielded 3 negative associations, for delays of 0, 2, and 3 months (TE=0.027, 0.039, and 0.032 bits, respectively, and ρ= −0.222, −0.352, and −0.265, respectively). It was marginally different from 0 for a delay of 1 month (*p*=0.013) and significantly different from 0 for delays of 2 and 3 months (*p*=0.002 and 0.006, respectively).

Results for TE from firearm restrictiveness to individual components of deaths per firearm owner are reported in Figure 2e–i and Appendix Table 4 (available online). TE to firearm ownership unveiled 3 positive associations for delays of 0, 1, and 3 months, all significantly different from 0 (TE=0.040, 0.031, and 0.028 bits, respectively; *p*=0.001, 0.006, and 0.011, respectively; and ρ=0.198, 0.244, and 0.229, respectively). TE to firearm deaths, firearm accidents, and firearm homicides did not reveal any associations, positive or negative. Instead, TE to firearm suicides yielded negative associations marginally different from 0 for delays of 2, 4, and 5 months (TE=0.024, 0.029, and 0.036 bits, respectively; *p*=0.022, 0.012, and 0.004, respectively; and ρ=−0.004, −0.047, and −0.099, respectively).

**Figure 3:**
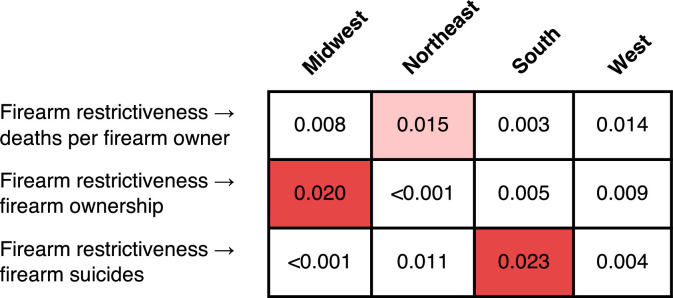
Results for analyses on a regional level. Each row reports the amount of transfer entropy computed for the analysis list on the left. Transfer entropy from firearm restrictiveness to deaths per firearm (top row) was evaluated with a delay of 3 months, transfer entropy to firearm ownership (middle row) was evaluated with a delay of 0 months, and transfer entropy to firearm suicides (bottom row) was evaluated with a delay of 2 months. Red and pink cells indicate a negative causal association that is different from 0 with a significance level of α=0.05 and α=0.01, respectively.

Results for regional analyses are reported in Figure 3. TE from firearm restrictiveness to deaths per firearm owner was computed with a delay of 3 months. It uncovered a negative association in the Northeast that is marginally different from 0 (TE=0.015 bits, *p*=0.085, and ρ= −0.084). TE from firearm restrictiveness to firearm ownership was computed with a delay of 0 months and revealed a negative association in the Midwest that is significantly different from 0 (TE=0.020 bits, *p*=0.038, and ρ= −0.128). Finally, because no associations were detected for TE from firearm restrictiveness to firearm deaths, but 3 were found to firearm suicides, the authors computed the latter in each region for a delay of 5 months. A negative association that is significantly different from 0 was uncovered in the South (TE=0.023 bits, *p*=0.028, and ρ= −0.052).

## DISCUSSION

In this study, the authors introduce an information-theoretic approach to study the influence of firearm legislation on firearm ownership and firearm harms, simultaneously. The authors sought to elucidate whether a restrictive (permissive) legal landscape would reduce (increase) the safety of owning a firearm, quantified as the number of deaths per firearm owner. In agreement with their prediction, the authors found that restrictive firearm laws led to a safer environment with lower deaths per firearm owner. The effect was observed immediately and lasted for 3 additional months. This finding supports the conclusions of several studies in literature^31^^,^^34^^,^^38^^,^^39^^,^^48^^,^^49^ and is the first to demonstrate the role of restrictive legislation in reducing firearm harms systematically, across law classes and U.S. states. Notwithstanding, the result was further digested to draw additional conclusions.

In contrast to their expectations, the authors found that accidents, homicides, and suicides per firearm owner all decreased in an increasingly restrictive firearm environment, albeit to different extents. Whereas the effect on firearm homicides and suicides lasted for 4 months, the effect on accidents lasted for 1 month only. It is tenable that certain classes of firearm laws address firearm deaths by intent differently.^20^^,^^35^^,^^38^^,^^39^ For example, Crifasi et al.^38^ stipulated that the effects of comprehensive background checks, permit-to-purchase, right-to-carry, and stand-your-ground laws impact firearm homicide but not suicides and accidents. It can be argued that certain law classes impact firearm accidents in the shorter term only^39^; however, research on the circumstances that lead to such disparate effects is required.

In subsequent assessment of the influence of firearm restrictiveness on firearm ownership, the authors found a positive association where restrictive laws led to greater rates of firearm ownership. The effect was observed immediately and lasted for the 3 succeeding months. This finding is consistent with existing literature related to panic buying, a well-documented phenomenon where crowds anticipate future scarcity of a product and buy unusually large amounts of it. It has been previously proposed that firearm regulation prompts firearm and ammunition sales among newly ineligible persons.^40^^,^^41^ In fact, panic buying of firearms was recorded in 2008 after the election of President Barack Obama, whose political agenda advocated for stricter firearm laws.^50^ Similar surges were observed in 2013 in New Jersey after Governor Christie's proposal to expand background checks and in Maryland after the ban of semiautomatic rifles.^50^ Thus, there is mounting evidence for panic buying of firearms.

Upon examination of the influence of firearm restrictiveness on firearm deaths, the authors discovered that firearm laws impact death rates differentially. The authors expected an effect parallel to the one observed in firearm ownership, yet restrictive firearm laws did drive down firearm deaths. When disaggregated by intent, only firearm suicides were impacted by the legal environment. This result suggests that the observed influence of firearm laws on firearm safety is largely driven by increasing the prevalence of firearms and, to a lesser extent, by decreasing firearm suicides. The authors also explored the possibility of reverse causal effects where firearm restrictiveness is influenced by the outcomes. The authors discovered a negative association between deaths per firearm owner and firearm restrictiveness. When the authors disaggregated the measure of deaths per firearm owner into its components, they found that this relationship is driven by a positive association between firearm ownership and firearm restrictiveness. This finding further supports the notion of panic buying preceding the implementation of restrictive firearm laws.

In addition to analyses on a national level, the authors investigated the impacts of regional legal environments on firearm safety, firearm ownership, and firearm deaths. Only in the Northeast did legislation impact deaths per firearm owner, following the national pattern. This finding could be explained by regional legal landscapes. In the Northeast, 65 firearm-related laws were passed with only 6 of them being permissive, corresponding to 9.23% of the laws. When comparing this figure with 20.69% in the West, 38.46% in the South, and 49.33% in the Midwest, a pattern emerges where the more restrictive laws are passed, the safer citizens are. Furthermore, only in the Midwest did the implementation of restrictive laws reduce firearm ownership, contrary to the trend found on a national level. It is plausible that citizens in the Midwest were unaware of the restrictive laws passed^51^ or that they did not perceive them as strict.^52^ Research on citizens’ interpretation of individual firearm laws and their perception of threat on their ability to bear arms could shed light on this finding. Finally, upon examination of the influence of firearm restrictiveness on firearm suicides, only the South exhibited a negative response. Because no influence was observed on a national level, it is possible that the large proportion of deaths in this region (twice as in the Midwest and West and 3 times as in the Northeast) dominated the analysis on a national level.

### Limitations

This study is among the first to examine the role of firearm legislation on firearm harms in conjunction with firearm ownership. In a country where the right to bear arms is enshrined in its constitution, it is crucial to understand the influence of firearm legislation on both outcomes toward the formulation of agreeable policies. Nonetheless, this study is not free of limitations. First, the international collaboration within this study prevents the use of the National Center for Health Statistics’ restricted data such that mortality counts below 10 are suppressed. Moreover, the authors consider only firearm deaths as a measure of firearm harms, although the rates of firearm injuries are substantially greater than those of firearm fatalities.^53^^,^^54^ Firearm injuries also pose non-negligible costs to the American economy, estimated at 557 billion dollars annually.^3^^,^^54^, ^55^, ^56^ For complete assessment of firearm harms, one could analyze data on firearm injuries, made available through the Centers for Disease Control and Prevention WISQARS database.^57^ However, WISQARS does not return results on state and month resolutions, thereby limiting data-driven methodologies. Moreover, firearm injuries are usually underreported,^58^^,^^59^ and the intent of injury is difficult to determine owing to the illicit nature of firearm violence.^60^^,^^61^ Thus, inclusion of firearm injuries in quantitative analyses remains a challenge.

Another limitation relates to the granularity of the analyses and their implication for state legislation. The authors conducted tests on national and regional levels, which do not inform on the effects of firearm laws in individual state, nor could they elucidate the intricate roles of state demographics and culture. Geographic disparities likely stem from differences in laws, urban, demographics, economics, and culture in the U.S. regions and states.^6^^,^^56^^,^^62^ However, approaches that infer causality on the basis of information theory or dynamical systems require rich time series with variation, and the majority of states have implemented only a few firearm-related laws.^44^ Consequently, these methods become nonviable on a state level. Ultimately, firearm research presents a need for methodologic advances in causal inference methods that can address single-point interventions while systematically accounting for multiple treatments with potentially identical outcomes that may be taking place (nearly) simultaneously.

## CONCLUSIONS

The authors present evidence for the role of restrictive legislation in promoting safe firearm ownership. Within an information-theoretic framework, the authors demonstrate that after the implementation of restrictive firearm laws, firearm acquisition rates considerably increase, and at the same time, firearm deaths nominally reduce. The effects vary with respect to death by intent and geographic locality. The results provide a first understanding of how firearm laws might impact firearm harms and ownership simultaneously. This study should be expanded upon with granular analyses to provide insights into the roles of demographics, socioeconomics, and culture to inform effective legislation that minimizes firearm harms while allowing law-abiding citizens to bear arms.

## CRediT authorship contribution statement

**Roni Barak Ventura:** Conceptualization, Methodology, Software, Validation, Formal analysis, Investigation, Data curation, Writing – original draft, Writing – review & editing, Visualization, Project administration, Funding acquisition. **James Macinko:** Conceptualization, Writing – review & editing, Funding acquisition. **Manuel Ruiz Marín:** Methodology, Software, Validation, Formal analysis, Investigation, Writing – review & editing, Funding acquisition. **Maurizio Porfiri:** Conceptualization, Methodology, Software, Validation, Formal analysis, Investigation, Resources, Writing – review & editing, Supervision, Project administration, Funding acquisition.

## Appendix Supplementary materials

Image, application 1
