# Supplementary material for: Association of State Firearm Laws With Firearm Ownership and Mortality
Source: AJPM Focus. 2024 Jun 20;3(4):100250. doi: 10.1016/j.focus.2024.100250 (PMC11301056; doi:10.1016/j.focus.2024.100250)
Supplement: Supplementary file 1 [file mmc1.pdf]

Supplementary Information

Appendix Section 1. Time series processing

Data were collected for each month between January 2000 and October 2019 on state and national levels with no incomplete points. In order to achieve a granular analysis on a regional level, data state-level data were aggregated into the four U.S. regions (Appendix Figure 1) based on U.S. Census Bureau designations [1]. Specifically, the Northeast included Connecticut, Maine, Massachusetts, New Hampshire, New Jersey, New York, Pennsylvania, Rhode Island, and Vermont; The Midwest included Illinois, Indiana, Iowa, Kansas, Michigan, Minnesota, Missouri, Nebraska, North Dakota, Ohio, South Dakota, and Wisconsin; The South included Alabama, Arkansas, Delaware, Florida, Georgia, Kentucky, Louisiana, Maryland, Mississippi, North Carolina, Oklahoma, South Carolina, Tennessee, Texas, Virginia, and West Virginia; and the West included Arizona, California, Colorado, Idaho, Montana, Nevada, New Mexico, Oregon, Utah, Washington, and Wyoming.

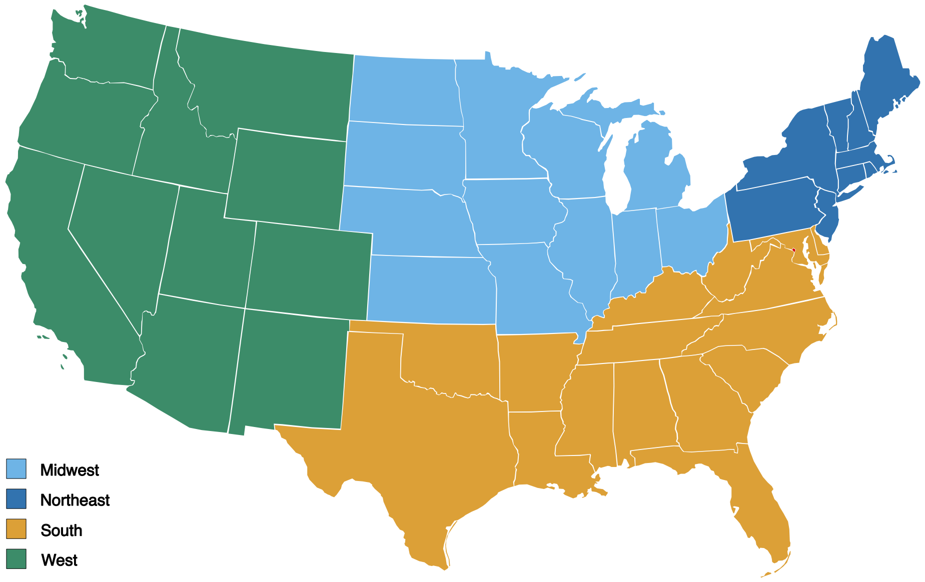

Appendix Figure 1: The four U.S. regions, as defined by the U.S. Census Bureau.

Of the 318 legislative actions, 89 were passed in the South, 87 in the West, 77 in the Midwest, and 65 in the Northeast (Appendix Figure 2a). Correspondingly, the majority of firearm deaths were recorded in the South (292,910), followed by the West (140,597), Midwest (135,026), and Northeast (68,706) (Appendix Figure 2b). Firearm ownership was most prevalent in the South where an average of 62.4 million firearms were recorded (Appendix Figure 2c). Firearm ownership was equally prevalent in the Midwest and West with an average of 31.4 million and 32.4 million firearms, respectively, and least prevalent in the Northeast with an average of 17.4 million firearms. Also for processed time series, data were fully generated with no incomplete points (Appendix Figure 3). Firearm restrictiveness in both the Midwest and West contained 50 changes whereas 54 and 43 changes were observed in the South and Northeast, respectively.
In total, we generated ten time series on national and regional levels: firearm restrictiveness, firearm ownership, firearm deaths, firearm homicides, firearm suicides, deaths per firearm owner, accidents per firearm owner, homicides per firearm owner, and suicides per firearm owner.

Seasonal adjustment and detrending
The time series we generated exhibited seasonality and/or lacked stationarity. Using them in their raw form in an information-theoretic framework would give rise to incorrect inference of interactions. Thus, we seasonally-adjusted

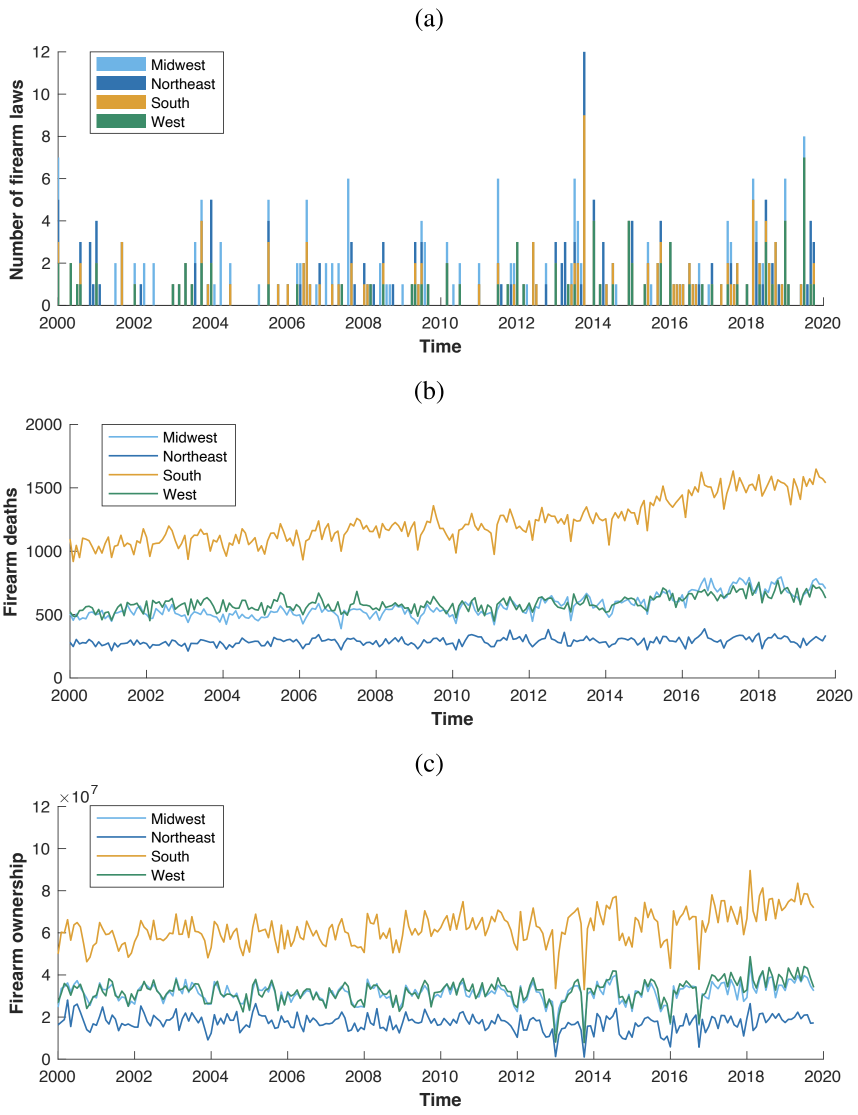

Appendix Figure 2: Raw regional times series collected for this study. The time series included the number of firearm laws (a), firearm deaths (b), and firearm ownership (c) for each region. In all plots, the Midwest, Northeast, South, and West are represented in light blue, blue, yellow, and green colors, respectively.

(a)

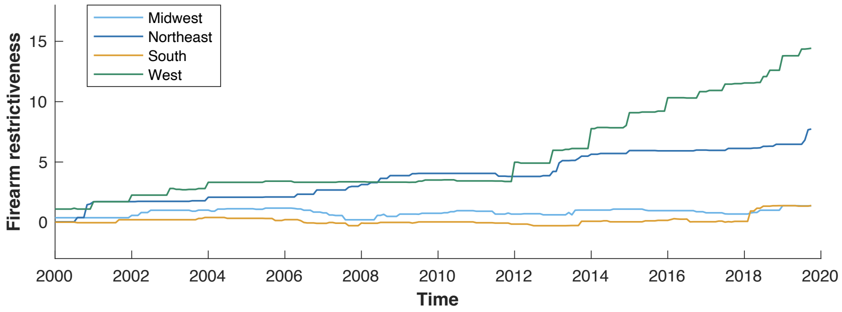

(b)

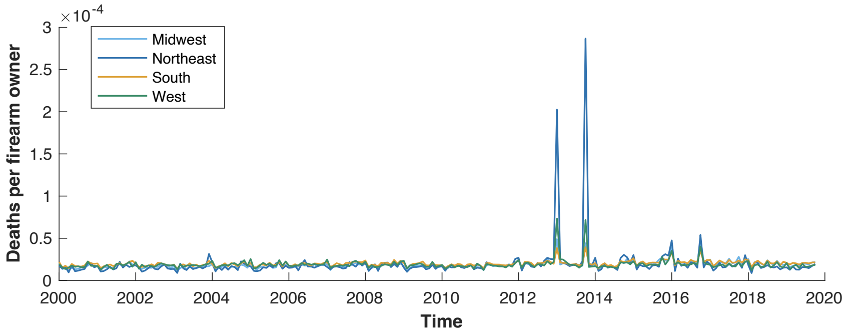

Appendix Figure 3: Processed time series of (a) firearm restrictiveness and (b) deaths per firearm owner in the Midwest (light blue), Northeast (blue), South (yellow), and West (green).

the time series using the forecast [2] package on R (version 8.18). We applied the msts function for periods of 3, 4, 6, and 12, followed by the mstl function to decompose the time series into seasonal, trend cycle, and irregular components. Following this procedure, the augmented Dickey–Fuller test was used to ensure the stationarity of the seasonally-adjusted and detrended time series (Appendix Table 1).

Before seasonal adjustment and detrending, firearm ownership was stationary in the Northeast ($p = 0.038$) but it was not stationary in the Midwest ($p = 0.212$), South ($p = 0.345$), and West ($p = 0.202$), nor on a national level ($p = 0.252$). Considering the raw death rates, time series were non-stationary nationally and across regions ($p > 0.349$). However, when divided by intent, the national and regional time series for firearm homicides and firearm suicides were non-stationary ($p > 0.156$ and $p > 0.355$, respectively), and firearm accidents were stationary ($p < 0.031$). Firearm restrictiveness was non-stationary on a national level ($p = 0.999$) as well as a regional level ($p = 0.762$, 0.999, 0.858, and 0.999 for the Midwest, Northeast, South, and West respectively). Time series for deaths per firearm owner were stationary in the nation and region scales ($p < 0.031$), except for the South ($p = 0.106$). Considering deaths per firearm owner by intent, accidents per firearm owner were stationary on a national ($p = 0.004$) and across regions ($p < 0.001$). Homicides per firearm owner and suicides per firearm owner were stationary on a national level ($p = 0.036$ and $p = 0.026$, respectively) and across regions except for the South ($p = 0.092$ and $p = 0.101$, respectively). After seasonal adjustment and detrending, all time series were stationary ($p < 0.001$).

	National		Midwest		Northeast		South		West	
	before	after	before	after	before	after	before	after	before	after
**Firearm ownership**	0.252	< 0.001	0.212	< 0.001	0.038	< 0.001	0.345	< 0.001	0.202	< 0.001
**Firearm deaths**	0.558	< 0.001	0.502	< 0.001	0.349	< 0.001	0.566	< 0.001	0.424	< 0.001
**Firearm accidents**	0.031	< 0.001	< 0.001	< 0.001	< 0.001	< 0.001	0.006	< 0.001	< 0.001	< 0.001
**Firearm homicides**	0.445	< 0.001	0.322	< 0.001	0.156	< 0.001	0.443	< 0.001	0.253	< 0.001
**Firearm suicides**	0.569	< 0.001	0.478	< 0.001	0.355	< 0.001	0.552	< 0.001	0.413	< 0.001
**Firearm restrictiveness**	0.999	< 0.001	0.762	< 0.001	0.999	< 0.001	0.858	< 0.001	0.999	< 0.001
**Deaths per firearm owner**	0.031	< 0.001	0.019	< 0.001	< 0.001	< 0.001	0.106	< 0.001	0.002	< 0.001
**Accidents per firearm owner**	0.004	< 0.001	< 0.001	< 0.001	< 0.001	< 0.001	0.001	< 0.001	< 0.001	< 0.001
**Homicides per firearm owner**	0.036	< 0.001	0.016	< 0.001	< 0.001	< 0.001	0.092	< 0.001	0.004	< 0.001
**Suicides per firearm owner**	0.026	< 0.001	0.016	< 0.001	< 0.001	< 0.001	0.101	< 0.001	0.001	< 0.001

Appendix Table 1: Reported p-values for the augmented Dickey-Fuller test, assessing the stationarity of national and regional time series before and after seasonal adjustment and detrending.

Time series symbolization
To capture the time series' dynamics in a manner that is consistent across variables, we pursued a symbolic approach [3, 4]. For each variable, we transcribed its values to symbols reflect whether each value is smaller or greater than the median of the entire time series [5]. Monthly values larger than the median were assigned a value of 1 and values less than or equal to the median 0. As such, the symbolized time series indicated whether the variable assumed a high or low value at a given time step t.

Appendix Section 2. Transfer entropy analysis

Transfer entropy is a model-agnostic approach for the inference of temporal associations between pairs of time series. Extensions of this framework allow the inference of associations in the presence of nonlinear interactions, confounding variables, and time delays [6, 7, 8, 9]. It was successfully implemented in a wide range of applications, including neuroscience [6, 8, 10], climatology [11, 12], economics [13, 14], animal behavior [15, 16, 17], and human behavior [18, 19, 20].
Transfer entropy is based on Shannon entropy as a measure of uncertainty [21]. Assuming X is a discrete random variable with a sample space of $\mathscr{X}$, the entropy of X is computed as

$$H(X) = -\sum_{x\in\mathscr{X}} p(x)\log p(x), \tag{1}$$

where $p(x)$ is the probability that X assumes the value x. If a logarithm base of 2 is specified, then $H(X)$ is measured in bits. Given Equation 1, the joint entropy of random variables X and another discrete random variable Y can be defined as

$$H(X,Y) = -\sum_{x\in\mathscr{X},y\in\mathscr{Y}} p(x,y)\log p(x,y) \tag{2}$$

and their conditional entropy can be defined as

$$H(X|Y) = -\sum_{x\in\mathscr{X},y\in\mathscr{Y}} p(x,y)\log p(x|y), \tag{3}$$

where y is a realization of Y, with a sample space $\mathscr{Y}$. The joint entropy represents the overall uncertainty of both X and Y, whereas the conditional entropy can be interpreted as the amount of uncertainty of variable X, knowing the realization of Y.
Given Equation (2) and Equation (3), transfer entropy from Y to X is computed as

$$\text{TE}_{Y\to X} = H(X_{t+1}|X_t) - H(X_{t+1}|X_t,Y_t). \tag{4}$$

In this formulation, transfer entropy from variable X to variable Y quantifies the reduction of uncertainty in predicting the future state of Y, given knowledge about Y's present state and the present state of X [22]. If Y is independent from X and does not contain information that predict it, then $H(X_{t+1}|X_t,Y_t)$ will equal $H(X_{t+1}|X_t)$ and transfer entropy will be zero.
To take into account the possibility of a delay in the response of X to changes in Y, Equation 4 can be adapted to introduce a time lag. Specifically, we shift the time-series of Y relative to the time-series of X by δ [10]

$$\text{TE}_{Y\to X} = H(X_{t+1}|X_t) - H(X_{t+1}|X_t,Y_{t-\delta}). \tag{5}$$

In all analyses, we considered delays ranging from zero to eleven months $\delta \in \{0,1,...,11\}$.
To determine whether a value of transfer entropy is statistically different from chance, we compared it against a surrogate distribution [8]. For a given pair of variables X and Y, the time series of the source variable Y was randomly shuffled and transfer entropy was computed between the two. This quantity would represent the amount of transfer entropy that emerged by chance from a pair of uninteracting time series. We repeated this procedure 50,000 times to generate 50,000 values of transfer entropy, from which we constructed a surrogate distribution. We tested whether the observed transfer entropy was in the right tail of the surrogate distribution. If it had exceeded its 95th

percentile, then transfer entropy was deemed significantly different from zero. If it had exceeded its 90th percentile, then transfer entropy was deemed as a trend. In cases where our analysis considered multiple delays, the threshold percentile was corrected for multiple comparisons via False Discovery Rate [23].
When transfer entropy was found to be non-zero, we aimed to elucidate whether the association is positive or negative. To this end, we computed the partial correlation between the two seasonally adjusted, detrended, and not symbolized time series. If the partial correlation coefficient ρ was greater than zero, the association was deemed positive (X increases when Y increases and X decreases when Y decreases). In contrast, if the partial correlation coefficient was lower than zero, the association was deemed negative (X decreases when Y increases and X increases when Y decreases).

Appendix Section 3. Evaluating different measures of firearm prevalence

In our analysis, we used a measure of deaths per firearm owner that was computed as the number of firearm deaths, divided by firearm ownership. Firearm ownership is a measure of firearm prevalence that was formulated in [24]. This model estimates the monthly fraction of firearm owners out of the population by integrating two cogent proxies, background checks per capita and fraction of suicides committed with a firearm, and calibrating on yearly survey data that assess ownership. It accounts for geographic spill over effects whereby firearms move across state borders and incorporates temporal autoregression.
Nonetheless, firearm prevalence can be evaluated through other means, namely background checks [25, 26] and fraction of firearm suicides [27, 28, 29, 30]. Although both measures intuitively approximate firearm prevalence, they have certain limitations. Background checks do not always realize into an acquisition, or could cover the multiple firearm purchases (especially in states with permit-to-purchase laws [31]). Moreover, private party sales and firearm show sales may not yield a background check as only licensed federal dealers are required to do so [26]. Similarly, firearm suicides are not always driven by accessibility to firearms. Women tend to choose less violent methods to commit suicides [32], and individuals in general may choose alternative means based on socioeconomic factors such as religion and honor culture [33, 34].
While background checks may be an effective proxy of firearm prevalence, in practice it approximates the rate at which firearms enter the public domain. That is, the raw number of background checks estimates the number of firearms that were potentially acquired during the time of sampling. In the interest of quantifying the total amount of firearms that are available to citizens, one could integrate the number of background checks over time. Here, we compare the results of our analysis when three alternative measures of firearm prevalence are used to compute deaths per firearm owner: background checks, integrated background checks, and fraction of firearm suicides.
Data on firearm ownership, the measure of prevalence we use to compute deaths per firearm owner in the main manuscript, was derived from a model described in [24] (Appendix Figure 4a). The model estimates the fraction of firearm owners in each state every month. Therefore, in order to approximate the number of firearms in each state every month, we multiplied the model's output by the state population size in the respective year. Although the obtained measurement reflects the number of firearm owners in a given state and month, it is the closest estimate of the number of firearm one could obtain in the absence of a national registry.
Data on background checks were collected from the Federal Bureau of Investigation's National Instant Criminal Background Check System (NICS) [35]. NICS was founded in November 1998 to execute the Brady Handgun Violence Prevention Act, mandating authorized firearm vendors to submit background check requests for prospective buyers. To approximate the number of acquired firearms, counts of permits for "Handgun", "Long Gun", "Other", and "Multiple" firearms were aggregated. Background checks administered for permit re-checks, pawns, redemptions, and rentals were excluded as they do not correspond to newly acquired firearms. In the specified time period, a minimum of 364 thousand background checks were performed in June 2002 and a maximum of 2.16 million in December 2012 (Appendix Figure 4b). Since the number of background checks reflects the addition of newly owned firearm and not the cumulative number of firearms owned, we also considered the integral of background checks. From the time series of background checks, we created time series of its cumulative sum since the initiation of NICS (Appendix Figure 4c). Overall, this time series reaches a maximum of 200 million background checks that were performed nationwide.
Finally, we computed the fraction of firearm suicides. This variable was computed as the number of suicides committed with firearms out of all suicides committed with any means. In order to compute this measure, we

collected the total number of suicides from CDC's Wonder Underlying Cause of Death database [36]. Specifically, we collected the number of deaths by "Injury Intent and Mechanism". To obtain the number of suicides committed with firearms, we specified "Suicide" as the injury intent and "Firearm" as the injury mechanism. To obtain the number of all suicides (with firearms and otherwise), we specified "Suicide" as the injury intent and "All Causes of Death" as the injury mechanism. Both queries were grouped the results by state, year, and month to obtain the monthly time series. For each month, we took the number of firearm suicides (collected as aforementioned above) and divided it by the total number of suicides. In order to obtain the estimated number of firearms, we multiplied each fraction by the state population size in the respective year. In the time period between January 2000 and October 2019, the number of firearms estimated from the fraction of firearm suicides was lowest in September 2014 with a count of 143 million firearms and reached its highest value of 177 million in January 2018 (Appendix Figure 4d).
From the four measures of firearm prevalence, we computed four different measures of deaths per firearm owner. Specifically, we divided the number of firearm deaths in a given month of a given year by the measure of prevalence in the corresponding month, and generated four time series: deaths per firearm owner, deaths per background checks, deaths per integrated background checks, and deaths per firearm suicides. To quantify the influence of firearm restrictiveness on each of these measures, we computed transfer entropy between the pair of time series.
Negative associations were detected from firearm restrictiveness to firearm deaths per firearm owner, for delays of zero, one, two, and three months (TE=0.031, 0.022, 0.033, and 0.038 bits and $\rho = -0.239$, -0.271, -0.371, and -0.271, respectively; Appendix Table 2). For delays of zero, two, and three months, transfer entropy was significantly different from zero ($p = 0.007$, 0.005, and 0.003, respectively), whereas it was marginally different from zero for a delay of one month ($p = 0.030$). No associations were detected for any of the alternative measures of firearm prevalence (deaths per background checks, deaths per integrated background checks, and deaths per fraction of firearm suicides). The improved performance of the model's output relative to the alternative measures of firearm prevalence likely lies in its ability to capture multiple dimensions of firearm ownership and use. The authors suggest that inclusion of additional firearm ownership measures could improve the estimates of firearm ownership, however, the introduction of additional variables into the model would undermine its power. Thus, the model is limited to the most commonly used proxies of firearm prevalence.

Appendix Section 4. Results of transfer entropy analyses and partial correlations

For complete reporting of our transfer entropy analyses, we include tables summarizing the amount of transfer entropy we computed, the p-values of permutation tests, and the partial correlation coefficients for all delays (zero to eleven months). Appendix Table 3 contains the results for transfer entropy from firearm restrictiveness to deaths per firearm owner disaggregated by intent, whereas Appendix Table 4 contains the results for transfer entropy from firearm restrictiveness to firearm ownership and to firearm deaths disaggregated by intent.

Appendix Section 5. Testing for reverse associations

Our study analyzes directional associations from firearm restrictiveness to deaths per firearm owner, firearm ownership, and firearm mortality. It is tenable that reverse associations exist whereby firearm restrictiveness is influenced by deaths per firearm owner, firearm ownership, and firearm mortality. For example, Luca et al. have demonstrated a 15% increase in firearm bills following the occurrence of a mass shooting.[37] Appendix Table 5, contains the results for transfer entropy where firearm restrictiveness serves as the target variable, rather than the source variable.
Transfer entropy from deaths per firearm owner to firearm restrictiveness revealed a negative association that was significantly different from zero for a delay of eight months (TE=0.051 bits, $p < 0.001$, and $\rho = -0.239$) and two positive associations that are marginally different from zero for delays of one and three months (TE=0.029 and 0.023 bits; $p = 0.008$ and $p = 0.023$; and $\rho = 0.298$ and 0.103, respectively). Transfer entropy from firearm ownership to firearm restrictiveness indicated a negative association that is significantly different from zero for a delay of one month (TE=0.036 bits, $p = 0.003$, and $\rho = -0.308$) and a positive association for a delay of eight months (TE=0.060 bits, $p < 0.001$, and $\rho = 0.128$). No associations were identified for transfer entropy from firearm deaths to firearm restrictiveness.

Similar to our key findings, associations between deaths per firearm owner and firearm restrictiveness appear to be driven by changes in firearm ownership, rather than firearm deaths. The increase in firearm ownership eight months before restrictive legislation is implemented could reflect panic-buying of firearms when the intent to pass a restrictive law is announced to the public. However, detailed research on the circumstances that could lead to a decrease in firearm ownership one and three months prior to legislation is warranted.

(a)

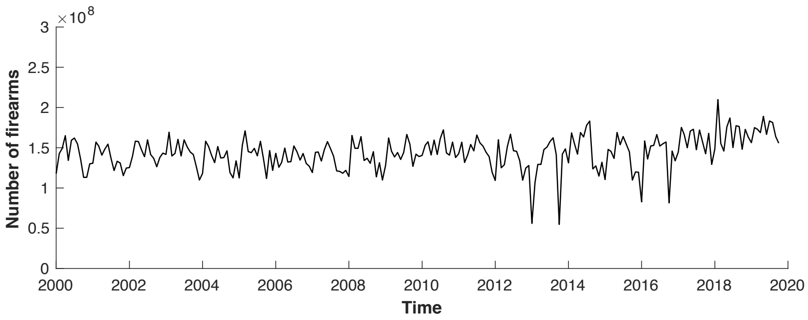

(b)

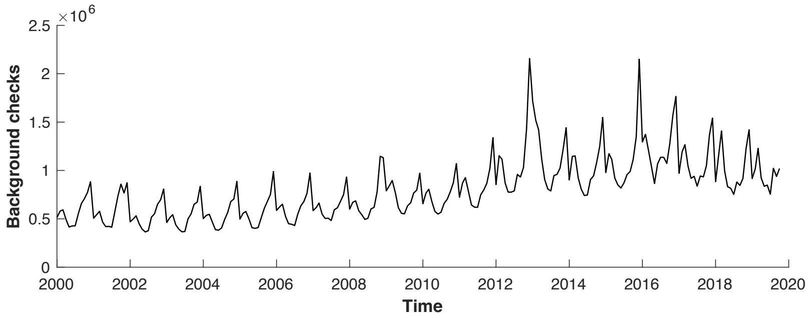

(c)

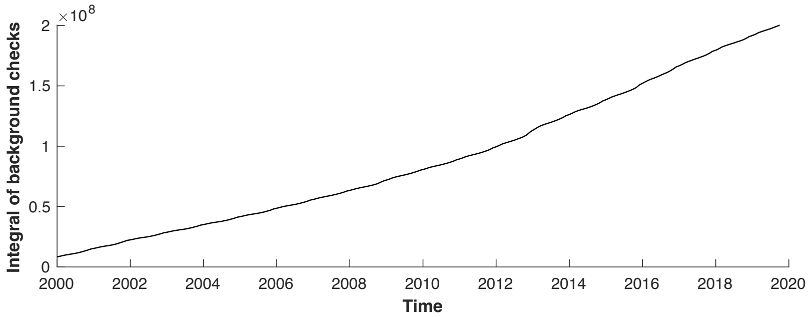

(d)

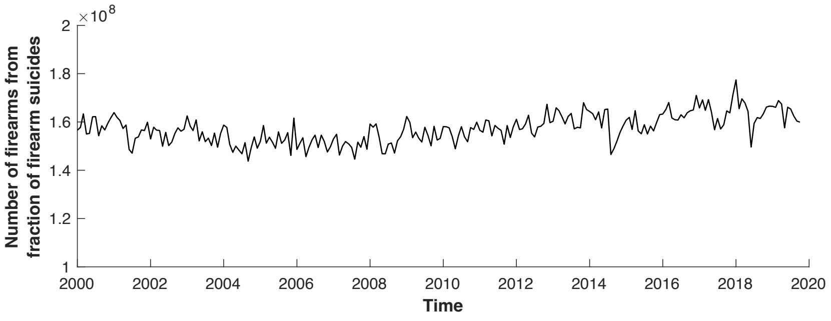

Appendix Figure 4: Raw time series of four measures of firearm prevalence used in this analysis: (a) firearm ownership, (b) background checks, (c) integral of background checks, and (d) fraction of firearm suicides.

Target \ Delay	0	1	2	3	4	5	6	7	8	9	10	11
**deaths per firearm owner**	0.031<br>*0.007*<br>(−0.239)	0.022<br>*0.030*<br>(−0.271)	0.033<br>*0.005*<br>(−0.371)	0.038<br>*0.003*<br>(−0.271)	< 0.001<br>*0.999*<br>(−0.059)	0.008<br>*0.301*<br>(0.066)	< 0.001<br>*0.969*<br>(0.302)	0.005<br>*0.532*<br>(0.273)	0.005<br>*0.479*<br>(0.332)	0.002<br>*0.750*<br>(0.245)	0.007<br>*0.402*<br>(0.080)	0.012<br>*0.253*<br>(−0.022)
**Deaths per background check**	0.004<br>*0.530*<br>(0.068)	0.019<br>*0.045*<br>(0.013)	0.006<br>*0.410*<br>(−0.029)	0.005<br>*0.430*<br>(−0.115)	0.013<br>*0.127*<br>(−0.162)	0.001<br>*0.875*<br>(−0.169)	0.008<br>*0.305*<br>(−0.067)	0.006<br>*0.435*<br>(0.028)	0.001<br>*0.938*<br>(0.052)	0.008<br>*0.351*<br>(0.066)	0.005<br>*0.533*<br>(0.085)	0.001<br>*0.907*<br>(0.129)
**Deaths per integrated background checks**	< 0.001<br>*0.877*<br>(−0.051)	0.004<br>*0.509*<br>(−0.089)	0.019<br>*0.046*<br>(−0.140)	0.014<br>*0.107*<br>(−0.096)	0.006<br>*0.417*<br>(−0.008)	0.015<br>*0.107*<br>(0.060)	0.003<br>*0.631*<br>(0.124)	0.023<br>*0.038*<br>(0.137)	0.019<br>*0.074*<br>(0.148)	0.004<br>*0.586*<br>(0.144)	0.001<br>*0.895*<br>(0.115)	0.009<br>*0.360*<br>(0.010)
**Deaths per fraction of firearm suicides**	0.004<br>*0.526*<br>(0.019)	0.007<br>*0.321*<br>(−0.147)	0.015<br>*0.093*<br>(−0.176)	0.008<br>*0.267*<br>(−0.161)	0.019<br>*0.051*<br>(−0.155)	< 0.001<br>*0.996*<br>(< 0.001)	0.004<br>*0.544*<br>(0.070)	0.018<br>*0.072*<br>(0.089)	0.005<br>*0.494*<br>(0.136)	0.002<br>*0.770*<br>(0.143)	0.003<br>*0.651*<br>(0.187)	0.020<br>*0.095*<br>(0.092)

Appendix Table 2: Transfer entropy from firearm restrictiveness (source) to four possible measures of firearm deaths per firearm prevalence (target). Each interaction was study with delays ranging from zero to eleven months. Within each cell, the top value represents the amount of transfer entropy. The middle value in italics represents the p-value of a permutation test assessing whether transfer entropy is significantly different from zero with a significance level of $\alpha = 0.05$ and a trend level of $\alpha = 0.1$. The bottom value in parentheses represents the partial correlation coefficient. Red and pink entries indicate a negative association that is different from zero, after correcting significance and trend levels for multiple comparisons using the False Discovery method
.

Target \ Delay	0	1	2	3	4	5	6	7	8	9	10	11
**deaths per firearm owner**	0.031<br>*0.007*<br>(−0.239)	0.022<br>*0.030*<br>(−0.271)	0.033<br>*0.005*<br>(−0.371)	0.038<br>*0.003*<br>(−0.271)	< 0.001<br>*0.999*<br>(−0.059)	0.008<br>*0.301*<br>(0.066)	< 0.001<br>*0.969*<br>(0.302)	0.005<br>*0.532*<br>(0.273)	0.005<br>*0.479*<br>(0.332)	0.002<br>*0.750*<br>(0.245)	0.007<br>*0.402*<br>(0.080)	0.012<br>*0.253*<br>(−0.022)
**Accidents per firearm owner**	0.034<br>*0.004*<br>(−0.211)	0.024<br>*0.020*<br>(−0.225)	0.002<br>*0.700*<br>(−0.291)	0.007<br>*0.322*<br>(−0.155)	0.012<br>*0.151*<br>(−0.095)	0.001<br>*0.837*<br>(−0.058)	0.003<br>*0.650*<br>(0.150)	0.005<br>*0.493*<br>(0.178)	0.003<br>*0.631*<br>(0.292)	0.001<br>*0.943*<br>(0.185)	0.003<br>*0.739*<br>(0.168)	0.007<br>*0.439*<br>(0.063)
**Homicides per firearm owner**	0.018<br>*0.056*<br>(−0.234)	0.026<br>*0.015*<br>(−0.268)	0.029<br>*0.010*<br>(−0.358)	0.039<br>*0.002*<br>(−0.249)	0.002<br>*0.786*<br>(−0.040)	0.007<br>*0.333*<br>(0.099)	0.007<br>*0.336*<br>(0.330)	0.001<br>*0.832*<br>(0.286)	0.001<br>*0.825*<br>(0.328)	0.004<br>*0.620*<br>(0.214)	0.003<br>*0.663*<br>(0.043)	0.018<br>*0.124*<br>(−0.075)
**Suicides per firearm owner**	0.027<br>*0.013*<br>(−0.222)	0.018<br>*0.058*<br>(−0.254)	0.039<br>*0.002*<br>(−0.352)	0.032<br>*0.006*<br>(−0.265)	< 0.001<br>*0.996*<br>(−0.057)	0.011<br>*0.194*<br>(0.057)	0.005<br>*0.500*<br>(0.273)	< 0.001<br>*0.954*<br>(0.247)	0.005<br>*0.513*<br>(0.307)	0.003<br>*0.683*<br>(0.239)	0.009<br>*0.306*<br>(0.085)	0.004<br>*0.625*<br>(0.001)

Appendix Table 3: Transfer entropy from firearm restrictiveness (source) to deaths per firearm owner disaggregated by intent (target). Each interaction was study with delays ranging from zero to eleven months. Within each cell, the top value represents the amount of transfer entropy. The middle value in italics represents the p-value of a permutation test assessing whether transfer entropy is significantly different from zero with a significance level of $\alpha = 0.05$ and a trend level of $\alpha = 0.1$. The bottom value in parentheses represents the partial correlation coefficient. Red and pink entries indicate a negative association that is different from zero, after correcting significance and trend levels for multiple comparisons using the False Discovery method
.

Target \ Delay	0	1	2	3	4	5	6	7	8	9	10	11
**Firearm ownership**	0.040<br>*0.001*<br>(0.198)	0.031<br>*0.006*<br>(0.244)	0.017<br>*0.063*<br>(0.313)	0.028<br>*0.011*<br>(0.229)	0.004<br>*0.573*<br>(0.037)	0.001<br>*0.841*<br>(−0.065)	0.006<br>*0.393*<br>(−0.250)	0.004<br>*0.599*<br>(−0.259)	0.006<br>*0.440*<br>(−0.257)	< 0.001<br>*0.959*<br>(−0.164)	0.005<br>*0.557*<br>(−0.009)	0.002<br>*0.763*<br>(0.054)
**Firearm deaths**	0.002<br>*0.775*<br>(0.042)	0.022<br>*0.032*<br>(−0.075)	0.008<br>*0.298*<br>(−0.113)	0.008<br>*0.294*<br>(−0.141)	0.012<br>*0.162*<br>(−0.075)	0.010<br>*0.235*<br>(−0.048)	< 0.001<br>*0.912*<br>(0.005)	0.004<br>*0.611*<br>(0.030)	0.006<br>*0.426*<br>(0.045)	0.003<br>*0.704*<br>(0.189)	0.008<br>*0.382*<br>(0.239)	0.010<br>*0.323*<br>(0.189)
**Firearm accidents**	0.014<br>*0.109*<br>(−0.083)	0.006<br>*0.355*<br>(−0.109)	0.008<br>*0.298*<br>(−0.172)	0.006<br>*0.380*<br>(−0.082)	0.017<br>*0.075*<br>(−0.139)	0.006<br>*0.423*<br>(−0.124)	0.010<br>*0.244*<br>(0.008)	0.004<br>*0.536*<br>(0.042)	0.009<br>*0.297*<br>(0.153)	0.001<br>*0.909*<br>(0.087)	0.005<br>*0.570*<br>(0.193)	0.005<br>*0.566*<br>(0.144)
**Firearm homicides**	0.005<br>0.490<br>(−0.027)	0.015<br>*0.089*<br>(−0.090)	0.015<br>*0.087*<br>(−0.146)	0.014<br>*0.117*<br>(−0.096)	< 0.001<br>*0.978*<br>(−0.056)	0.004<br>*0.533*<br>(0.050)	0.004<br>*0.586*<br>(0.132)	0.020<br>*0.062*<br>(0.089)	0.001<br>*0.863*<br>(0.102)	0.018<br>*0.093*<br>(0.081)	0.024<br>*0.051*<br>(0.068)	0.966<br>*0.969*<br>(−0.030)
**Firearm suicides**	0.006<br>*0.403*<br>(0.086)	0.001<br>*0.799*<br>(0.010)	0.024<br>0.022<br>(−0.004)	< 0.001<br>*0.974*<br>(−0.100)	0.029<br>0.012<br>(−0.047)	0.036<br>0.004<br>(−0.099)	0.003<br>*0.643*<br>(−0.114)	0.002<br>*0.738*<br>(−0.078)	0.012<br>*0.208*<br>(−0.041)	0.001<br>*0.868*<br>(0.181)	< 0.001<br>*0.906*<br>(0.257)	0.016<br>*0.149*<br>(0.278)

Appendix Table 4: Transfer entropy from firearm restrictiveness (source) to firearm ownership and firearm deaths, disaggregated by intent (target). Each interaction was study with delays ranging from zero to eleven months. Within each cell, the top value represents the amount of transfer entropy. The middle value in italics represents the p-value of a permutation test assessing whether transfer entropy is significantly different from zero with a significance level of $\alpha = 0.05$ and a trend level of $\alpha = 0.1$. The bottom value in parentheses represents the partial correlation coefficient. Blue entries indicate positive associations that are different from zero, after correcting significance levels for multiple comparisons using the False Discovery method. Pink entries indicate a negative trend, after correcting significance levels for multiple comparisons
.

Source \ Delay	0	1	2	3	4	5	6	7	8	9	10	11
**deaths per firearm owner**	0.010<br>*0.184*<br>(0.202)	0.029<br>*0.008*<br>(0.298)	0.003<br>*0.582*<br>(0.254)	0.023<br>*0.023*<br>(0.103)	< 0.001<br>*0.956*<br>(0.069)	0.001<br>*0.861*<br>(−0.085)	0.006<br>*0.419*<br>(−0.162)	0.005<br>*0.506*<br>(−0.208)	0.051<br>*<0.001*<br>(−0.154)	0.017<br>*0.107*<br>(0.004)	0.016<br>*0.136*<br>(0.046)	0.005<br>*0.577*<br>(0.170)
**Firearm ownership**	0.018<br>*0.058*<br>(−0.200)	0.036<br>*0.003*<br>(−0.308)	< 0.001<br>*0.999*<br>(−0.234)	0.021<br>*0.035*<br>(−0.099)	0.003<br>*0.573*<br>(−0.018)	< 0.001<br>*0.977*<br>(0.106)	0.001<br>*0.795*<br>(0.176)	< 0.001<br>*0.967*<br>(0.209)	0.060<br>*<0.001*<br>(0.128)	0.007<br>*0.401*<br>(0.007)	0.012<br>*0.216*<br>(−0.067)	0.004<br>*0.630*<br>(−0.177)
**Firearm deaths**	0.001<br>*0.767*<br>(−0.026)	0.001<br>*0.765*<br>(−0.012)	0.001<br>*0.868*<br>(−0.024)	0.009<br>*0.230*<br>(−0.001)	0.003<br>*0.628*<br>(−0.033)	0.001<br>*0.774*<br>(−0.018)	0.001<br>*0.799*<br>(−0.045)	0.012<br>*0.186*<br>(−0.014)	0.004<br>*0.593*<br>(0.066)	0.005<br>*0.538*<br>(0.088)	0.002<br>*0.792*<br>(0.081)	0.014<br>*0.195*<br>(0.060)

Appendix Table 5: Transfer entropy from deaths per firearm owner, firearm ownership and firearm deaths (source) to firearm restrictiveness (target). Each interaction was study with delays ranging from zero to eleven months. Within each cell, the top value represents the amount of transfer entropy. The middle value in italics represents the p-value of a permutation test assessing whether transfer entropy is significantly different from zero with a significance level of $\alpha = 0.05$ and a trend level of $\alpha = 0.1$. The bottom value in parentheses represents the partial correlation coefficient. Blue and red entries indicate positive and negative associations that are different from zero, after correcting significance levels for multiple comparisons using the False Discovery method. Pink entries indicate a negative trend, after correcting significance levels for multiple comparisons
.

References

1. U.S. Census Bureau. 2013 Census Regions and Divisions of the United States. URL https://www2.census.gov/geo/pdfs/maps-data/maps/reference/us$_{r}egdiv.pdf; Date accessed : 2023 - 01 - 04$.
2. Hyndman RJ, Khandakar Y. Automatic time series forecasting: The forecast package for R. *J Stat Softw*. 2008; 27(1): 1–22. DOI: 10.18637/jss.v027.i03.
3. Staniek M, Lehnretz K. Symbolic transfer entropy. *Phys Rev Lett*. 2008; 100(15): 158101.
4. Porfiri M, Ruiz Marin M. Symbolic dynamics of animal interaction. *J Theor Biol*. 2017; 54: 145–156.
5. Porfiri M, Barak-Ventura R, Marín MR. Self-protection versus fear of stricter firearm regulations: examining the drivers of firearm acquisitions in the aftermath of a mass shooting. *Patterns*. 2020; 1(6): 100082. DOI: 10.1016/j.patter.2020.100082.
6. Vicente R, Wibral M, Lindner M, Pipa G. Transfer entropy: A model-free measure of effective connectivity for the neurosciences. *J Comput Neurosci*. 2011; 30(1): 45–67. DOI: 10.1007/s10827-010-0262-3.
7. Ito S, Hansen ME, Heiland R, Lumsdaine A, Litke AM, Beggs JM. Extending transfer entropy improves identification of effective connectivity in a spiking cortical network model. *PLoS One*. 2011; 6(11): e27431. DOI: 10.1371/journal.pone.0027431.
8. Bossomaier T, Barnett L, Harré M, Lizier JT. 2016. An introduction to transfer entropy: Information flow in complex systems (Springer International Publishing, New York, NY).
9. Papana A, Papana-Dagiasis A, Siggiridou E. Shortcomings of transfer entropy and partial transfer entropy: Extending them to escape the curse of dimensionality. *Int J Bifurc Chaos*. 2020; 30(16): 2050250. DOI: 10.1142/S0218127420502508.
10. Wibral M, Pampu N, Priesemann V, et al. Measuring information-transfer delays. *PLoS One*. 2013; 8(2): e55809. DOI: 10.1371/journal.pone.0055809.
11. Runge J, Heitzig J, Petoukhov V, Kurths J. Escaping the curse of dimensionality in estimating multivariate transfer entropy. *Phys Rev Lett*. 2012; 108(25): 258701. DOI: 10.1103/PhysRevLett.108.258701.
12. Runge J, Heitzig J, Marwan N, Kurths J. Quantifying causal coupling strength: A lag-specific measure for multivariate time series related to transfer entropy. *Phys Rev E*. 2012; 86(6): 061121. DOI: 10.1103/PhysRevE.86.061121.
13. Marschinski R, Kantz H. Analysing the information flow between financial time series. *Eur Phys J B*. 2002; 30(2): 275–281. DOI: 10.1140/epjb/e2002-00379-2.
14. Papana A, Kyrtsou C, Kugiumtzis D, Diks C. Detecting causality in non-stationary time series using partial symbolic transfer entropy: Evidence in financial data. *Comput Econ*. 2016; 47(3): 341–365. DOI: 10.1007/s10614-015-9491-x.
15. Orange N, Abaid N. A transfer entropy analysis of leader-follower interactions in flying bats. *Eur Phys J Spec Top*. 2015; 224(17-18): 3279–3293. DOI: 10.1140/epjst/e2015-50235-9.
16. Porfiri M. Inferring causal relationships in zebrafish-robot interactions through transfer entropy: A small lure to catch a big fish. *Anim Behav Cogn*. 2018; 5(4): 341–367.
17. Doi I, Deng W, Ikegami T. Spontaneous and information-induced bursting activities in honeybee hives. *Sci Rep*. 2023; 13(1): 11015. DOI: 10.1038/s41598-023-37785-8.
18. Borge-Holthoefer J, Perra N, Gonçalves B, et al. The dynamics of information-driven coordination phenomena: A transfer entropy analysis. *Sci Adv*. 2016; 2(4): e1501158. DOI: 10.1126/sciadv.1501158.
19. Nakayama S, Marín MR, Camacho M, Porfiri M. Plasticity in leader-follower roles in human teams. *Sci Rep*. 2017; 7(1): 14562. DOI: 10.1038/s41598-017-14851-6.
20. Barak Ventura R, Richmond S, Nadini M, Nakayama S, Porfiri M. Does winning or losing change players' engagement in competitive games? Experiments in virtual reality. *IEEE Trans Games*. 2019; 13(1): 23–34. DOI: 10.1109/TG.2019.2928795.
21. Shannon CE. A mathematical theory of communication. *Bell Labs Tech J*. 1948; 27(3): 379–423. DOI: 10.1002/j.1538-7305.1948.tb01338.x.
22. Schreiber T. Measuring information transfer. *Phys Rev Lett*. 2000; 85(2): 461–464.
23. Benjamini Y, Hochberg Y. Controlling the false discovery rate: a practical and powerful approach to multiple testing. *J R Stat Soc Series B Stat Methodol*. 1995; 57(1): 289–300. DOI: 10.1111/j.2517-6161.1995.tb02031.x.
24. Barak-Ventura R, Marín MR, Porfiri M. A spatiotemporal model of firearm ownership in the United States. *Patterns*. 2022; 3(8): 100546. DOI: 10.1016/j.patter.2022.100546.
25. Porfiri M, Sattanapalle RR, Nakayama S, Macinko J, Sipahi R. Media coverage and firearm acquisition in the aftermath of a mass shooting. *Nat Hum Behav*. 2019; 3(9): 913–921. DOI: 10.1038/s41562-019-0636-0.
26. Wallace LN. Responding to violence with guns: Mass shootings and gun acquisition. *Soc Sci J*. 2015; 52(2): 156–167. DOI: 10.1016/j.soscij.2015.03.002.
27. Miller M, Azrael D, Hemenway D. Firearm availability and unintentional firearm deaths, suicide, and homicide among 5–14 year olds. *J Trauma Acute Care Surg*. 2002; 52(2): 267–275. DOI: 10.1097/00005373-200202000-00011.
28. Reeping PM, Cerdá M, Kalesan B, Wiebe DJ, Galea S, Branas CC. State gun laws, gun ownership, and mass shootings in the US: cross sectional time series. *Br Med J*. 2019; 364: l542. DOI: 10.1136/bmj.l542.
29. Nagin DS. Firearm availability and fatal police shootings. *Ann Am Acad Pol Soc Sci*. 2020; 687(1): 49–57. DOI: 10.1177/0002716219896259.
30. Schell TL, Cefalu M, Griffin BA, Smart R, Morral AR. Changes in firearm mortality following the implementation of state laws regulating firearm access and use. *Proc Natl Acad Sci*. 2020; 117(26): 14906–14910. DOI: 10.1073/pnas.1921965117.
31. Webster D, Crifasi CK, Vernick JS. Effects of the repeal of Missouri's handgun purchaser licensing law on homicides. *Journal of Urban Health*. 2014; 91: 293–302.
32. Denning DG, Conwell Y, King D, Cox C. Method choice, intent, and gender in completed suicide. *Suicide and Life-Threatening Behavior*. 2000; 30(3): 282–288.
33. Brown RP, Imura M, Osterman LL. Gun culture: Mapping a peculiar preference for firearms in the commission of suicide. *Basic and Applied Social Psychology*. 2014; 36(2): 164–175.

34. Kõlves K, McDonough M, Crompton D, De Leo D. Choice of a suicide method: Trends and characteristics. *Psychiatry Research*. 2018; 260: 67–74.
35. Federal Bureau of Investogation. 2023 NICS Firearm Checks: Month/Year by State. URL https://www.fbi.gov/file-repository/nics$_{f}irearm_{c}hecks_{-m}onth_{y}ear_{b}y_{s}tate.pdf/view; Dateaccessed : 2023-01-04$.
36. Centers for Disease Control and Prevention WONDER. 2021 Underlying cause of death, 1999-2019. URL https://wonder.cdc.gov/ucd-icd10.html; Date accessed: 2023-01-04.
37. Luca M, Malhotra D, Poliquin C. The impact of mass shootings on gun policy. *J. Public Econ.*. 2020; 181: 104083. DOI: 10.1016/j.jpubeco.2019.104083.
